# Mouse models for post-sepsis syndrome: a comparison of cecal slurry injection model versus two cecal ligation and puncture models

**DOI:** 10.3389/fmed.2026.1774517

**Published:** 2026-05-22

**Authors:** John-Matthew K. Ang, Stephanie F. Mori, Erick Lewis, Maria E. C. Bruno, Arnold J. Stromberg, Hiroshi Saito

**Affiliations:** 1 Department of Surgery, University of Kentucky College of Medicine, Lexington, KY, United States; 2 Department of Pharmacology and Nutritional Sciences, University of Kentucky College of Medicine, Lexington, KY, United States; 3 Department of Statistics, University of Kentucky College of Arts and Sciences, Lexington, KY, United States; 4 Department of Physiology, University of Kentucky College of Medicine, Lexington, KY, United States

**Keywords:** cecal ligation and puncture (CLP), cecal slurry, chronic critical illness (CCI), debridement, murine models, post-intensive care syndrome (PICS), post-sepsis syndrome (PSS), sepsis

## Abstract

**Background:**

Sepsis survivors commonly suffer from chronic Post-Sepsis Syndrome (PSS). To understand the mechanisms of PSS, it is critical to utilize appropriate animal models. While cecal ligation and puncture (CLP) is a widely used model of sepsis, a concern exists regarding its suitability for studying long-term outcomes. Alternatively, we previously optimized a highly reproducible murine cecal slurry (CS)-injection model of sepsis. The objective of this study was to compare the suitability of CS and CLP models for investigating PSS.

**Methods:**

Male 4-month-old C57BL/6 mice were divided into 7 groups: (1) Naïve control; (2) Sham: laparotomy control; (3) CL: cecal ligation control without puncture; (4) CLP: conventional cecal ligation and puncture (21Gx2); (5) CLP-D: CLP followed by surgical removal (i.e., debridement) of the ligated distal cecum; (6) CS: cecal slurry injection; and (7) vehicle injection control for CS. All mice except for Naïve, received repeated antibiotics and fluid resuscitation starting at 12 h after infection or the control procedure. Blood samples were collected at 6 h and 1-month for IL-6 ELISA. A month later, all mice were euthanized, and qRT-PCR was performed to assess IL-6 gene expression in several tissues including the cecum.

**Results:**

Equivalent sepsis severity was confirmed in the sepsis groups (CS, CLP, and CLP-D) by similar survival rates (73–78%) and acute 6 h-plasma IL-6 levels (63.3–159.6 pg./mL). A month later, large abscesses, deformed necrotic tissues, and aberrant adhesions were frequently present in and around the cecum of CL, CLP, and CLP-D mice. Elevated levels of plasma IL-6 (>25 pg./mL) 1-month after sepsis induction were observed in both the CL and CLP groups and also in the CLP-D group to a lesser degree. Strong IL-6 gene expression was found only in the ligated distal cecum from the CLP and CL groups. The cecum abnormality and high levels of plasma IL-6 were completely absent in the CS group.

**Conclusion:**

The CLP model is accompanied with abnormal cecum which persistently produces IL-6. The CLP-D model with debridement reduces such problems to some extent but not completely. Because such experimental artifacts are absent in the CS model, this model appears to be better suited for studying PSS.

## 1 Introduction

Sepsis is a life-threatening organ dysfunction caused by a dysregulated host response to infection (1). Despite a consistent decrease in the sepsis mortality rate over the last 30 years, the sepsis incidence rate is increasing by nearly 13% annually. Consequently, the number of discharged sepsis survivors has more than doubled over the last decade, reaching well over 1.7 million new sepsis survivors annually (2, 3). Sepsis survivors commonly suffer from post-sepsis syndrome (PSS) or chronic critical illness (CCI) that significantly impairs their quality of life. Physical and cognitive dysfunction in sepsis survivors are widely recognized problems that have long-term effects on discharged sepsis patients (4–6). Studies now indicate that the vast majority of sepsis survivors report prolonged skeletal muscle weakness that persists years after recovery (7–10). Cognitive impairment affects up to 50% of sepsis survivors (11), with a majority of sepsis survivors reporting sepsis-associated delirium and over 80% of elderly survivors suffering from reduced functional states (12).

Although post-sepsis dysfunction has been widely recognized as a serious medical issue, the scientific community has historically lacked an animal model of sepsis that induces long-term dysfunction to identify the responsible mechanisms. We recognized that these previous limitations were a result of the following dilemma: animal models of sepsis are either too severe and cause early death of most animals without recovery from sepsis, or they are too mild and thus do not trigger long-term chronic dysfunction. To overcome this, we took major efforts to refine a non-surgical murine model of polymicrobial abdominal sepsis, whereby infection is initiated by injection of cecal slurry (CS), followed by fluid resuscitation and broad-spectrum antibiotic treatment which are purposefully delayed for 12 h or 24 h until animals become very sick (13). For this purpose, we previously developed a new CS preparation method using a glycerol/saline buffer, allowing the slurry to be frozen for at least 1 year without loss of bacterial viability. Because a large stock can be prepared in a single batch from multiple donor animals and dispensed into cryovials, this model ensures high reproducibility by eliminating batch-to-batch variability (13, 14). This clinically relevant resuscitation protocol allows for the development of sepsis with bacteremia, systemic inflammation, and organ damage in all mice, yet it rescues a majority of animals from an otherwise lethal condition and triggers long-term dysfunction such as skeletal muscle weakness in survivors (15, 16).

Cecal ligation and puncture (CLP) is a widely used rodent model for investigating sepsis; however, there is concern regarding whether this model is appropriate for studying long-term consequences after sepsis. This concern arises from unpublished observations from our earlier studies (17, 18) in which animals that survived CLP contained unresolved large amounts of necrotic tissue derived from the ligated cecum. Notably, such large necrotic tissue does not normally occur in sepsis patients who are discharged from the ICU. Earlier studies on rats with CLP models often included debridement to surgically remove the gangrenous ligated portion of the cecum to control the infection source (19, 20). However, the vast majority of newer studies, except for a few (21), omit this debridement procedure. Whether the above-mentioned concern in CLP models is truly problematic for a model of PSS and whether debridement after CLP can effectively eliminate this potential concern have not been experimentally examined previously. Therefore, in the present study we compared the CS injection model and two types of CLP models, with or without debridement, for their suitability in studies on post-sepsis chronic illness.

## 2 Materials and methods

### 2.1 Animals

Adult male C57BL/6 mice at age of 15-weeks were obtained from The Jackson Laboratory, and acclimated for at least 10 days but no longer than 21 days prior to experimentation. Thus, all mice underwent experiments at age of 4 months old. Animals were housed 4–5 animals per intra-ventilated (PIV) cages under controlled temperature (21–23 °C), humidity (30–70%), and lighting (14/10 light/dark cycle) with free access to drinking water and chow (Teklad Global No. 2918, 18% Protein Rodent Diet, Madison WI). All animal handling techniques in the present study were included in our Animal Use Protocol #2009–0541 which was approved by the Institutional Animal Care and Use Committee (IACUC) at the University of Kentucky and was in accordance with the National Institutes of Health guidelines for ethical treatment.

### 2.2 Experimental groups and overall procedure

Mice were divided into seven experimental groups. (1) Naïve control (Naïve; *n* = 8); (2) Sham control (Sham; *n* = 7); (3) Cecal ligation control (CL; *n* = 8); (4) Cecal ligation and puncture (CLP; *n* = 13); (5) Cecal ligation and puncture followed by debridement of the ligated portion of the cecum (CLP-D; *n* = 9); (6) Cecal slurry injection (CS; *n* = 11); and (7) Vehicle injection control (Vehicle; *n* = 7). Comparison of these experimental groups is summarized in Table 1.

**Table 1:** Description of procedures for each experimental group.

Group	Experimental description	Anesthesia and laparotomy	Cecal ligation	Cecal puncture	Debridement of ligated cecum	Post-surgery analgesia	Abdominal injection	Antibiotics and fluid resuscitation
Naïve	Non-sepsis control with no treatment	**−**	**−**	**−**	**−**	**−**	**−**	**−**
Sham	Non-sepsis control with laparotomy	**+**	**−**	**−**	**−**	**+**	**−**	**+**
CL	Non-sepsis control with cecal ligation	**+**	**+**	**−**	**−**	**+**	**−**	**+**
CLP	Sepsis induced by cecal ligation and puncture	**+**	**+**	**+**	**−**	**+**	**−**	**+**
CLP-D	Sepsis induced by CLP followed by debridement	**+**	**+**	**+**	**+**	**+**	**−**	**+**
CS	Sepsis induced by cecal slurry injection	**−**	**−**	**−**	**−**	**−**	**+**	**+**
Vehicle	Non-sepsis control with vehicle injection	**−**	**−**	**−**	**−**	**−**	**+**	**+**

### 2.3 Cecal ligation and puncture (CLP), cecal debridement surgery, and other related control procedures

Mice in the CLP group received a standard CLP procedure with a method similar to our previous studies with a few minor modifications (17, 18). A small midline abdominal incision was performed on deeply anesthetized mice (by inhalation of 2.5% isoflurane in oxygen), and the cecum was ligated at 1 cm from the distal end with 5.0 synthetic absorbable sutures. This ligated portion was approximately 25–30% of the length of the entire cecum. The ligated portion of the cecum was gently squeezed and punctured twice with a 21-G needle. The cecum was then returned to the abdominal cavity, and the peritoneal muscle layer was closed with 5–0 synthetic absorbable sutures (every 4-mm) followed by separate closure of the skin with 5–0 non-absorbable sutures.

Mice in the CL control group received all the above procedures, including cecal ligation, but did not undergo puncture. Mice in the Sham control group received only the laparotomy portion of the CLP procedure outlined above. Mice in the CLP-D group first received all procedures identical to CLP followed by the secondary debridement procedure 24-28 h later. For this procedure, the abdominal incision was carefully reopened after which the entire portion of the ligated cecum was isolated and surgically resected. The resected site was disinfected by iodine application. Following debridement, the abdominal incision and skin were reclosed. After each surgery, all mice received analgesia by subcutaneous injection of Buprenorphine-SR (a sustained-release formulation of buprenorphine, 1.5 mg/kg) on the upper back. To avoid surgery-induced dehydration, 1 mL of warmed physiologic saline was given by subcutaneous injection in the lower back.

### 2.4 Preparation of cecal slurry (CS) stock

We have previously described both our original method (14) and an improved version (13) of preparing a frozen cecal slurry stock solution for sepsis study. In the present study, we added two modifications for further improvement. First, we further decreased the glycerol concentration from 10 to 5% (v/v) to minimize potential physiological stress, and second, we increased the number of straining mesh types from four to six in order to accelerate the straining process. Ceca were obtained from 30 donor male C57BL/6 mice at 4-months of age with an average body weight of 32.0 g. The cecal contents (a total 14.9 grams from the 30 mice) were collected, transferred into a sterile beaker, and initially suspended in 89-mL of 5% glycerol/saline by aggressive pipetting using 25-mL pipette.

To eliminate undigested fibrous food and swallowed hair, which later cause clogged injection needle when the slurry is administrated to mice, the slurry was passed through a series of six sterile mesh strainers with different mesh sizes (1910, 860, 380, 190, 140, and 74 μm in this order, Bellco Glass Inc., Vineland NJ) into the next sterile beaker. The two meshes (1910 and 140 μm) were newly included based on our previous observation that cecal slurry tended to clog the 860 and 74 μm meshes, thus slowing down the straining process. By inserting the 1910 μm and 140 μm meshes, the entire straining process went significantly faster. After each of the six straining processes, a further 10-mL of the same 5% glycerol/saline buffer was used to wash the remaining debris on the mesh. To collect the maximum amount of slurry, the cecal content was lightly pressed through the mesh by a sterile glass pestle (Bellco Glass Inc.). The total amount of glycerol/saline solution was 149-mL (89-mL for the initial suspension, and 10 mL x 6 time for washing), which follows our previous protocols recommending use of glycerol-saline solution at a ratio of 1-mL for every 100 mg wet cecal content weight. Following the final 74 μm filtration, the CS was maintained in a uniform suspension using a magnetic stirring bar in the 200-mL sterile glass beaker as previously described (14). While continuously stirred, 1-2-mL aliquots of cecal slurry were dispensed into cryovials and frozen slowly to −80 °C until use. A single batch (Lot: CS-190705) was used throughout the experiment eliminating variability from batch-to-batch differences or seasonal microbial shifts in donor animals.

### 2.5 Sepsis induction by cecal slurry (CS) injection

Since our cecal slurry (CS) is frozen with glycerol, almost 100% bacteria are preserved alive, allowing highly reproducible sepsis outcome (13–16). The frozen CS stock was thawed quickly in a 37 °C water bath and further incubated for an additional 10 min at 37 °C. Six hundred microliters of CS were injected into the peritoneal cavity of each mouse using a standard tuberculin syringe (25G × 16 mm). Our pilot study had confirmed that this dose of CS is 100% lethal to five mice if not resuscitated. Mice in the vehicle control group received the same volume of warm vehicle (5% glycerol in saline).

### 2.6 Resuscitation and monitoring

Twelve hours after sepsis induction (CLP, CLP-D, CS) or control procedures (Sham, CL, Vehicle), ICU-like resuscitation with antibiotics (imipenem 150 mg in 300 μL of saline, subcutaneous) and fluids (700 μL of saline, subcutaneous) were provided to each mouse every 12 h. Antibiotic treatment was continued for 5 days (total 9 times), and fluid resuscitation was continued until body temperature recovered to be at least 35.0 °C. This resuscitation procedure was very similar to our previous description (13, 15, 16) except that antibiotics were given subcutaneously on the back instead of intraperitoneally. This modification was to eliminate any possible effect from repeated intraperitoneal injections during the abdominal wound healing process in mice subjected to CLP. Survival was monitored daily for 1 month. Body weight was recorded before (Day 0) and after sepsis induction (daily, Day 1–7 and Day 14, 21, 28, and 28). Body temperature was also measured using a Thermocouple Meter (Model 20,250-91, Davis Instruments, Hayward, CA) with a rectal temperature probe (RET-3, Physitemp Instruments, Clifton, NJ) before (0 h) and after sepsis induction (6, 12, 24 h). On Day 28, whole body fat mass was monitored in each mouse using EchoMRI Body Composition Analyzer (EchoMRI LLC, Houston, TX, USA).

### 2.7 Euthanasia, tissue collection, and quantitative RT-PCR

Thirty days after sepsis induction or control procedures, animals were deeply anesthetized by inhalation of 2.5% isoflurane in oxygen, and euthanized by exsanguination while removing the blood from inferior vena cava (ivc). First, up to 1-mL of blood was collected from the ivc using a 26-G syringe needle, then the ivc was cut by scissors to ensure exsanguination. Wet tissue weights of the spleen and cecum (with stool content) were recorded. Photographs of the whole cecum were taken. As for the mice with ligated cecum (CL, CLP, and CLP-D groups), the cecum was carefully dissected and separated into the distal ligated portion and the proximal remnant portion. After the stool content was carefully removed, each cecal tissue was snap frozen in liquid nitrogen and stored at −80 °C. Additionally, the spleen, kidney, liver, and epididymal fat were also collected and snap frozen. The frozen tissues were later homogenized with TRIzol reagent (Invitrogen, Carlsbad, CA), total RNA was purified, and IL-6 mRNA levels were assessed as described previously (15).

### 2.8 Plasma sample collection and cytokine analysis

To assess both acute and chronic systemic inflammation, blood samples from each mouse were obtained at 6 h and 1-month after sepsis induction. For the 6 h blood collection, the mouse tail was nicked with a clean razor blade, and a small blood sample (10 μL) was collected from the tail vein. The blood was immediately mixed with 1 μL of 0.1 M sodium citrate to prevent coagulation. For 1-month blood collection, mice were anesthetized with isoflurane inhalation (2.5% in oxygen), and up to 1-mL of blood was collected from the inferior vena cava with 10% volume of 0.1 M sodium citrate. Plasma fractions were obtained from the collected blood samples through centrifugation (2,500xG for 15 min at 4 °C), and stored at −80 °C for later cytokine analysis by enzyme-linked immunosorbent assay (ELISA). For assessing acute inflammation at 6 h, a standard IL-6 ELISA kit (BMS603-2 Invitrogen) was used. Due to limited sample volume at 6 h, a few μL of plasma was diluted in the kit-provided buffer to meet the 50 μL assay requirement, and the final concentrations were calculated by multiplying the results by the dilution factor. For assessing chronic inflammation in 1-month plasma samples, a high sensitivity ELISA kit (eBIOSCIENCE, Vienna, Austria) was used without diluting the samples.

### 2.9 Statistical analysis

Sample sizes were determined based on prior experience with our previously published works using similar experimental paradigms (13, 15–18), where group sizes of *n* = 5–6 were sufficient to detect a 20% difference in biochemical and physiological outcomes with 80% power (*α* = 0.05). Accordingly, we enrolled 6–8 animals per non-sepsis control group and 9–13 per sepsis group (CLP, CLP-D, and CS). Following anticipated mortality during the study, final sepsis group sizes ranged from 7 to 10 animals, providing sufficient power to detect significant differences. Survival patterns were analyzed using Kaplan Meier LogRank test. For other experiments, One-way Analysis of Variance (ANOVA) tests were performed to compare the 7 experimental groups overall. Further, unpaired t-tests were performed for certain specific pairwise comparison between Sham versus CL, CLP, or CLP-D, CLP versus CS, and also Vehicle versus CS. For these analyses, two sample equal variance t-tests were used unless all measurements in a group were zero in which case a one sample t-test was used compared to zero. Throughout the study, a *p*-value of less than 0.05 was considered significant. All analyses were completed in JMP 16.0 (SAS Institute Inc.; Cary, NC, USA). Statistical analyses were performed by a statistician (AJS) in the University of Kentucky Department of Statistics.

## 3 Results

### 3.1 Survival rate after sepsis induction

**Figure 1:**
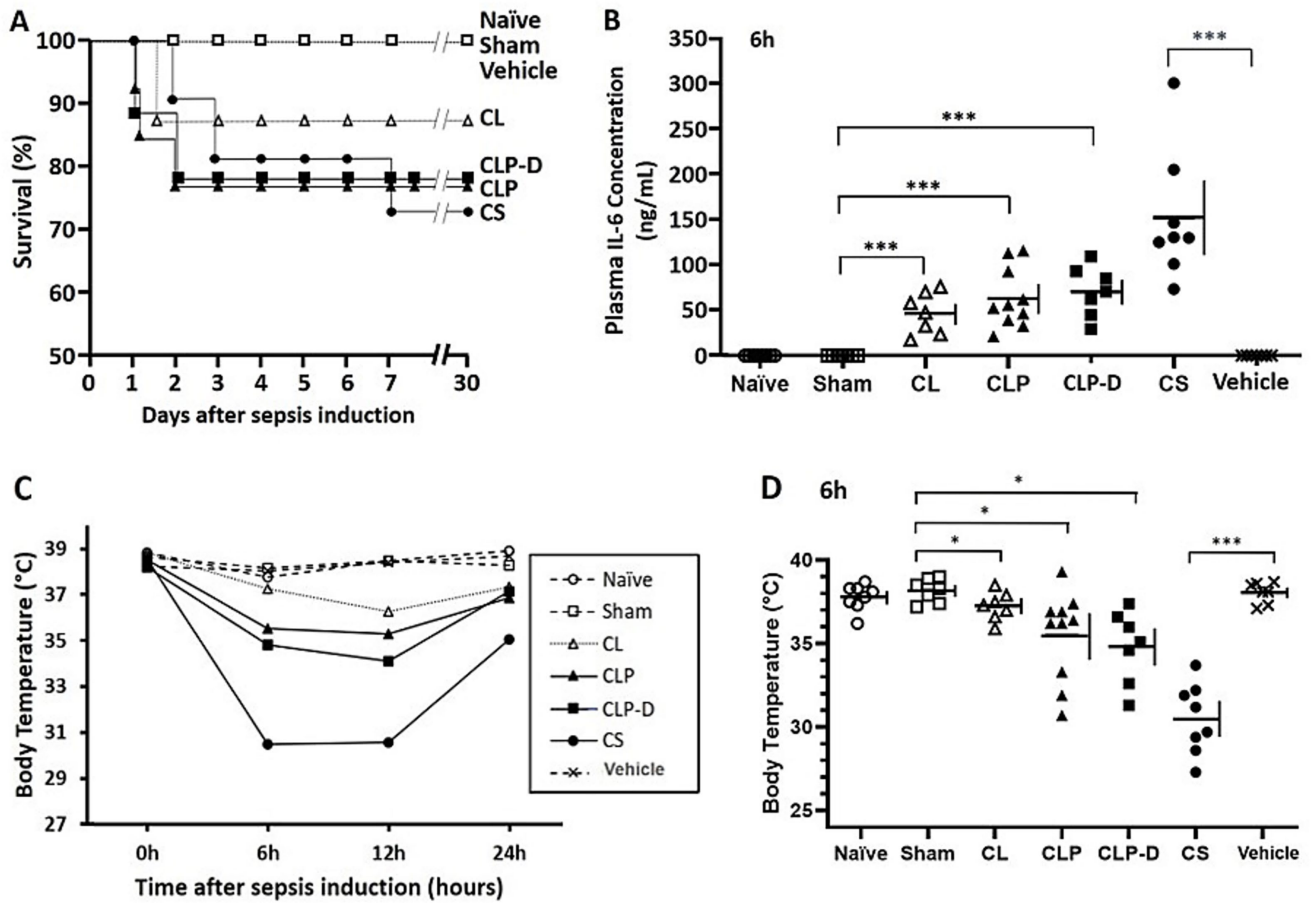
Mortality, acute IL-6 plasma levels, and hypothermia following experimental procedures. **(A)** Mouse survival after sepsis induction monitored for 1 month. Final survival rates for CL, CLP, CLP-D, and CS groups were 87.5, 76.9, 77.8, and 72.7%, respectively (*n* = 8–13 per group). No deaths occurred after Day 8, and no significant differences in survival patterns were observed among the four groups. **(B)** Plasma IL-6 concentrations 6 h post-sepsis induction. Note that CLP and CLP-D groups underwent the same procedure prior to this time-point. **(C)** Mean body temperature changes over 24 h. See panel **D** and Supplementary Figure S1 for standard deviations at each time point. **(D)** Individual mouse body temperature at 6 h; see Supplementary Figure S1 for other time points. For **B** and **D**, each symbol represents an individual mouse, and horizontal and vertical lines represent the mean and standard deviation, respectively. Only 1-month survivors are included **(B–D)**. Data were analyzed via one-way ANOVA (*p* < 0.0001) with pairwise t-tests for specific comparisons (*p < 0.05, and ***p < 0.001).

After sepsis induction (CLP, CLP-D, and CS) or control procedures (Naïve, Sham, CL, and Vehicle), each animal was monitored for survival for 30 days (Figure 1A). The three sepsis groups (CLP, CLP-D, and CS) showed similar survival rates (76.9, 77.8, and 72.7%, respectively). The CL control group also showed mortality, having an 87.5% survival rate. A LogRank test confirmed that there was no statistical difference among these four groups (CLP, CL, CLP-D, and CS). There was no mortality in Naïve, Sham surgery, and vehicle injection control groups.

### 3.2 IL-6 induction and hypothermia during acute phase

To compare the severity of acute systemic inflammation, circulating IL-6 levels were assessed from small blood samples obtained at 6 h after each procedure. As shown in Figure 1B, every mouse in the CL, CLP, CLP-D, and CS groups showed increases in 6 h plasma IL-6 levels (46.7, 56.4, 62.3, and 159.6 ng/mL in average, respectively), with the highest levels found in CS group. As expected, the CLP and CLP-D groups showed similar IL-6 levels at the 6 h time point, as mice in these two groups underwent identical procedures until the CLP-D mice received a debridement surgery next day. The three control groups (Naïve, Sham surgery, and Vehicle injection) did not show any detectable levels of IL-6 in the plasma (Figure 1B).

Since the degree of acute hypothermia serves as a good parameter for the severity of sepsis or acute systemic inflammation (17, 18), the rectal body temperature was monitored shortly before the procedures (0 h), and at 6, 12, and 24 h later (Figure 1C). Mice with CS-induced sepsis exhibited the most profound acute hypothermia at 6 h and 12 h post-injection. Two other sepsis groups, CLP and CLP-D, also showed prominent hypothermia. Mice in the CL group also showed significant, though less severe, hypothermia compared to three sepsis groups. After initiating resuscitation with fluids and antibiotics at 12 h, body temperature of these mice started to increase by 24 h. The three control groups (Naïve, Sham surgery, and Vehicle injection) did not show significant hypothermia. The time-course changes of each group’s average are presented in Figure 1C, while more detailed individual data with standard deviations are shown in Figure 1D (6 h) and Supplementary Figure S1 (0, 12, and 24 h).

### 3.3 Long-term changes in body weight, fat mass, and spleen weight

**Figure 2:**
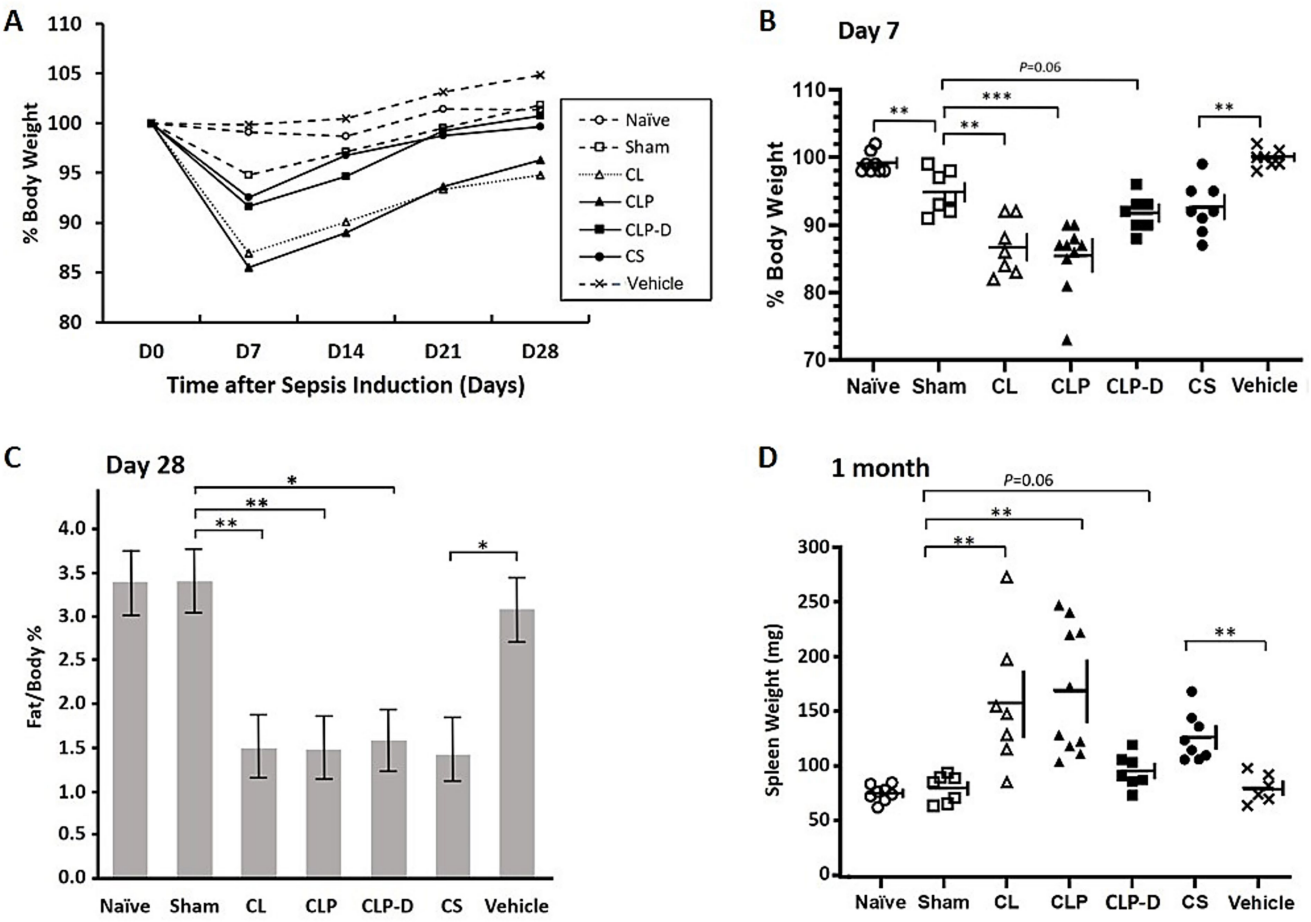
Long-term effects of sepsis and control procedures on body weight, body fat ratio, and spleen weight. Body weight for each mouse was monitored weekly and expressed as a percentage relative to the initial weight (Day 0). Data include only mice that survived for 1 month (*n* = 6–10 per group). **(A)** Average body weight change (%) over the time course normalized to Day 0. See panel **B** and Supplementary Figure S2 for standard deviations at each time point. **(B)** Relative body weight (%) of individual mice on Day 7. Data are shown as individual points with horizontal and vertical lines representing the mean and standard deviation, respectively. See Supplementary Figure S2 for other time points (Days 14, 21, and 28). **(C)** Body fat percentage 28 days after sepsis induction. Body fat percentages were measured by Echo-MRI for each living mouse 28 days after sepsis induction or control procedures. Each bar represents the mean and standard deviation of each group. **(D)** Spleen weight 1 month after sepsis induction. The whole wet tissue weight was recorded immediately after euthanasia. ANOVA showed *p* < 0.0001 **(A,B,D)** and *p* = 0.0002 **(C)**. Pairwise comparisons by *t*-tests also showed statistical significance as indicated by *, ** and *** (representing *p* < 0.05, *p* < 0.01, and *p* < 0.001, respectively). For **B** and **D**, horizontal and vertical lines represent the mean and standard deviation, respectively.

To assess chronic stress, body weight was monitored at least every week for 4 weeks (Figure 2A). Acute body weight loss was prominent by Day 7 in Sham, CL, CLP, CLP-D, and CS groups, where they lost an average of 5.2, 13.1, 14.5, 8.4, and 7.5% of their initial body weight, respectively. Mice with CLP and CL surgery developed the most profound weight loss by Day 7, which gradually recovered during the following weeks. However, significant weight loss remained at Day 28 in CLP and CL mice (an of average 4.7 and 5.2% body weight loss, respectively). CLP-D and CS groups showed less severe weight loss on Day 7 through Day 21, and nearly recovered their original weight by Day 28. Sham control mice showed modest weight loss by Day 7 that was resolved by Day 14 (Figures 2A,B; Supplementary Figure S2). The time-course changes of each group’s average weight are presented in Figure 2A, while detailed individual data with standard deviations are shown in Figure 2B (Day 7) and Supplementary Figure S2 (Days 14, 21, and 28).

As we previously reported, the loss of body fat is a major cause of weight loss after sepsis (15). Whole body fat ratios were assessed in each animal by Echo-MRI on Day 28 (Figure 2C). The three control groups (Naïve, Sham, and Vehicle) showed similar fat/body weight ratios (3.38, 3.40, and 3.07%, respectively). In contrast, the four groups (CL, CLP, CLP-D, and CS) that showed prominent weight loss, also showed significantly lower fat/body weight ratios (1.50, 1.49%. 1.58, and 1.48%, respectively, Figure 2C). Splenomegaly is routinely seen in mice that survived sepsis as we previously described (15). Thus, we compared the spleen weight immediately after mice were euthanized on Day 30. While all mice in the three control groups (Naïve, Sham, and Vehicle) had normal spleen sizes (<100 mg), all mice in the CS and CLP groups had significantly enlarged spleens (>100 mg). Nearly all CL mice, with one exception, also had enlarged spleens, similar to the CLP group. Modest splenomegaly was also found in some of the mice in CLP-D group (Figure 2D).

### 3.4 Long-term abnormalities of cecum after cecal ligation surgery

**Figure 3:**
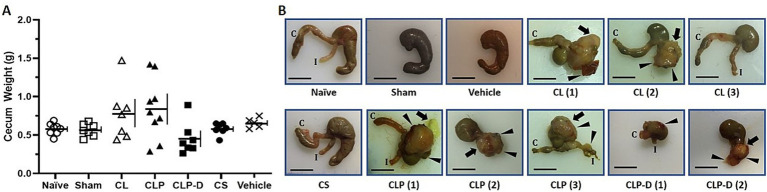
Cecal ligation causes long-term severe tissue abnormalities. **(A)** The whole cecum weight was recorded immediately after euthanasia 1 month after sepsis induction or control procedures. The weight includes the cecal tissue, contents (i.e., stool) and any abscesses present at the surgical site. The horizontal and vertical lines represent the mean and standard deviation, respectively. One-way ANOVA showed *p* = 0.0389. Pairwise comparisons by *t*-tests did not show statistical significance. **(B)** Representative images are shown for each group. Ceca from CL, CLP, and CLP-D mice exhibit severe abnormalities, including large abscess formation and adhesion to adjacent tissues. Black arrows indicate large abscesses or yellow pus leakage. Black arrowheads indicate portions of other tissues, such as liver or skin, attached to the abscess of the ligated cecum. Bar = 1 cm. C, Colon; I, Ileum.

The whole cecum size and shape were investigated in each mouse after euthanasia on Day 30 (Figure 3). The cecum weight of mice from Naïve, Sham, and Vehicle groups were within the normal range of 0.4 g – 0.75 g for 4-month-old male C57BL/6 mice as we described previously (14). The ceca from the CS group were also within this normal range, indicating that sepsis by the CS injection does not change the cecum size. Mice from the CL and CLP groups had widely varying cecum weights ranging from abnormally small (< 0.4 g) to abnormally large (> 0.8 g) up to nearly 1.5 g. Most of the CLP-D mice had smaller ceca as was expected due to the surgical removal of the ligated distal cecal portion. These results show that cecal ligation surgery alters cecum size while CS injection does not (Figure 3A).

Next, the cecum shape was compared across mice from all different groups (Figure 3B). Mice in the Sham and Vehicle control groups had ceca with normal appearance, similar to Naïve controls. All mice in CS-sepsis group also had normal ceca, indicating that this injection model of sepsis does not affect cecal shape. However, we occasionally observed modest peritoneal adhesions in the CS group, primarily involving mesenteric fat with the intestines or the abdominal wall (data not shown). In contrast, every cecum from CL, CLP, and CLP-D mice had abnormal shapes, particularly at the distal ligated area. As shown in Figure 3B, the abnormalities were mainly characterized by a large, round abscess filled with pus [CLP (1, 2, 3), CLP (1, 2), CLP-D (2)], and adhesion to surrounding tissues such as abdominal fat, liver [CL (1) and CLP (1)], other intestines, and skin [CLP (1)]. Though less frequently, some ceca showed a complete loss of the ligated portion in groups without debridement [CL (3)]. Mice with CLP-D showed mostly smaller ceca with a deformed remnant portion [CLP-D (1)], though an enlarged abscess was also seen in one case [CLP-D (2)]. These results indicate that cecal ligation alone causes dramatic cecal deformation, including a partial loss of cecal tissue, formation of large abscesses, and adhesion of the cecum to other tissues.

### 3.5 Persistent inflammation derived from the ligated cecum

**Figure 4:**
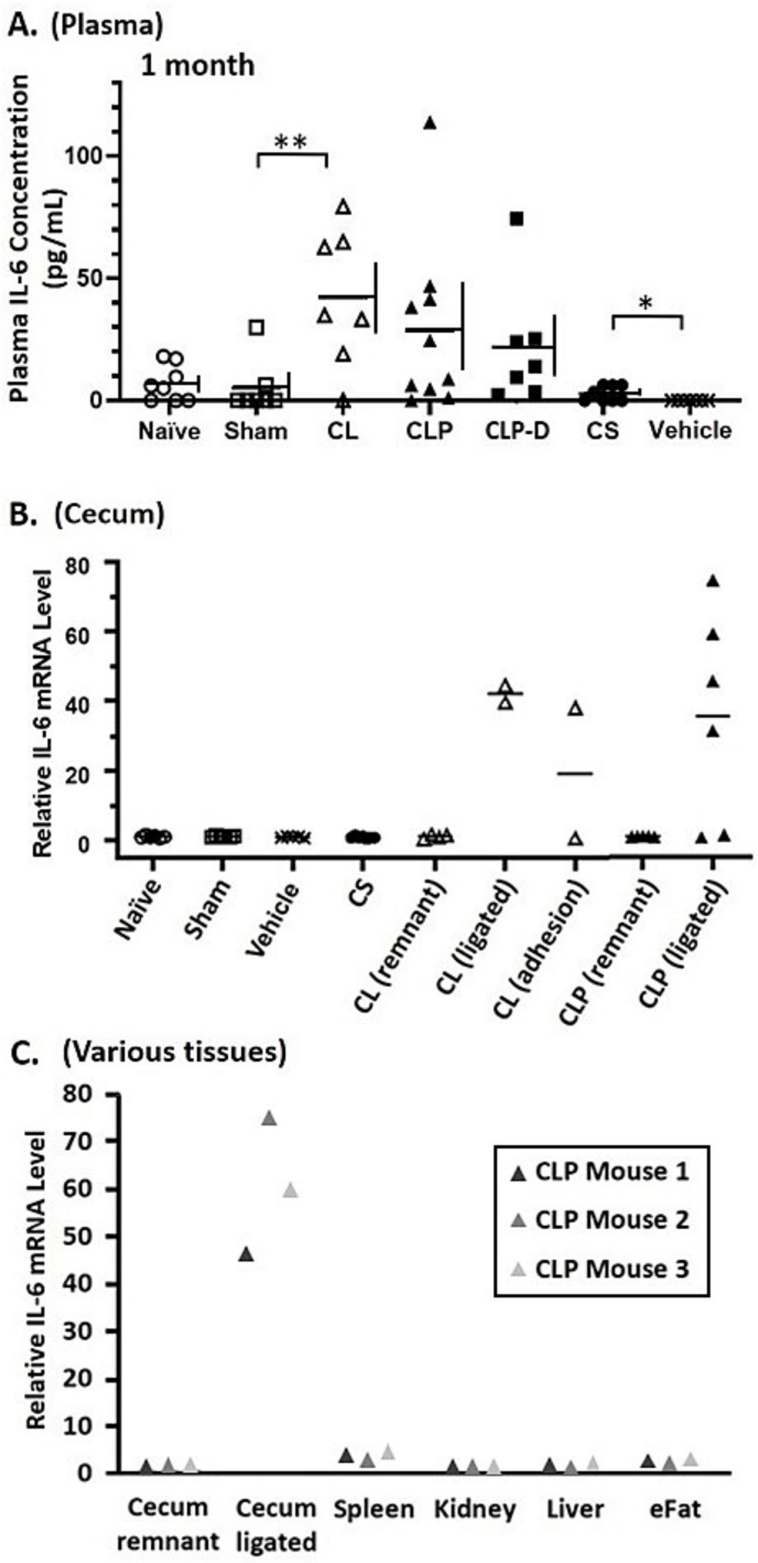
IL-6 expression analyses identify the ligated cecum as the primary source of sustained IL-6 production in CLP-induced sepsis survivor mice. All mice were euthanized 1 month after sepsis induction or control procedures, and blood and several tissues were collected immediately. **(A)** IL-6 concentrations in plasma from whole blood samples. One-way ANOVA showed *p* = 0.0016. Pairwise comparisons by *t*-tests also showed statistical significance (* and ** represent *p* < 0.05 and *p* < 0.01, respectively). Data are shown as individual points with horizontal and vertical lines representing the mean and standard deviation, respectively (*n* = 6–10 per group). **(B)** Levels of IL-6 mRNA in the cecum of each mouse. Some cecal tissues from CL and CLP groups were dissected and separated into the distal ligated portion (marked as “ligated”) and the remnant proximal portion (marked as “remnant”). A few cecal samples were collected with adherent skin or mesenteric tissues (marked as “adhesion”). All tissues from Naïve, Sham, Vehicle, and CS groups were whole ceca. **(C)** Levels of IL-6 mRNA in multiple tissues from three selected CLP mice that showed high levels of IL-6 production in **(A)**. The cecum tissues were separated into “remnant” and “ligated” as described in **(B)**. eFat, Epididymal fat tissue.

To assess chronic systemic inflammation, IL-6 concentrations were measured in the plasma samples obtained 30 days after the initial sepsis or control procedures. Plasma IL-6 levels in the Sham and Vehicle groups were comparable to Naïve controls. In the CL, CLP and CLP-D groups, however, most mice showed elevated plasma IL-6 concentrations compared to the Sham control group. The CS-sepsis group showed slightly elevated IL-6 levels compared to vehicle control group (Figure 4A). As the necrotic cecum was suspected to be a major source of sustained inflammation in these three groups (CL, CLP and CLP-D), IL-6 mRNA levels were compared in the ceca of these mice and compared to the control groups. IL-6 mRNA was nearly undetectable in the ceca of Naïve, Sham, Vehicle, and CS groups, consistent with their very low plasma IL-6 concentrations on Day 30. For the CLP, CL, and CLP-D groups, remnant ceca (i.e., the proximal portion of ligated ceca) showed very low IL-6 mRNA levels, while the ligated ceca (i.e., the distal portion of ligated ceca) mostly showed markedly high levels of IL-6 mRNA (Figure 4B).

Finally, to examine whether the ligated cecum is the major source of chronic IL-6 production in CLP mice, IL-6 mRNA levels were compared in the cecum with several other major tissues known to produce IL-6 (22, 23). When compared to other tissues, the ligated cecum expresses the highest IL-6 mRNA by far, including the remnant cecum, spleen, kidney, liver, and epididymal fat (Figure 4C). These results confirm that ligation of the cecum, not sepsis, causes persistent systemic inflammation with elevated levels of circulating IL-6 derived from the ligated distal cecum.

## 4 Discussion

The objective of the present study was to compare two major sepsis models, the cecal slurry injection model (CS model) and cecal ligation and puncture surgical model (CLP model), for their suitability for investigating chronic post-sepsis syndrome (PSS). For such a comparison, it was important to induce equivalently severe acute sepsis in each model. Thus, we carefully chose the dose of the CS injection and CLP puncture size so that the mortality rates of these models would be similar, thereby allowing for long-term comparison. To further align the biological conditions of the two models, we ensured that the CS donors and the CS recipient mice being identical in strain, sex, and age. Furthermore, we purchased these mice from a single vendor and maintained them under identical housing and acclimation conditions so that the CS group would be challenged with an autologous-like cecal microbiota, closely mimicking the endogenous infection of the CLP model. Since sepsis induction kinetics are different in these two models, multiple parameters such as acute IL-6 production, hypothermia, weight loss, and the final survival rate were measured to confirm similar sepsis severity across these two models. To enhance the clinical relevance of the current study, we included a resuscitation procedure with repeated antibiotics treatment and fluid administration, starting from 12 h after sepsis induction when all animals exhibit signs of systemic inflammation with hypothermia (13).

The 30-day time point was selected as the end-point of this study for two reasons. First, our previous work demonstrated that post-sepsis muscle weakness was equally profound at 14-and 70-days following sepsis induction (16), suggesting that the 30-day time point is a highly representative window for studying PSS. Second, this timeframe aligns with clinical practice, as 28-day mortality is a standard monitoring endpoint for ICU patients. The 30-day mortality rates of mice with CS injection and CLP surgery were similar with no statistical difference (27.3 and 23.1%, respectively). Acute hypothermia between 6 and 24 h was more profound in the CS group than CLP group, and acute IL-6 plasma levels at 6 h were higher in the CS group than CLP while acute loss rates were more profound in the CLP group than CS group (Figures 1, 2). Therefore, we confirmed that mice in the CS group had equivalent or slightly more severe acute sepsis compared to mice in the CLP group.

Despite the equivalent severity of sepsis during the acute phase, the CLP and CS sepsis models demonstrated different long-term inflammation. We found that about 50% of CLP mice exhibited elevated plasma IL-6 levels (>25 pg./mL) 1 month after sepsis induction, which was not observed in CS mice. Mice in other cecal surgery groups (CL and CLP-D) also had elevated chronic IL-6 levels (Figure 4A). Gene expression analysis identified the ligated portion of the cecum as the single major source of abnormal IL-6 production by mice after CLP (Figures 4B,C). We also found that the ligated portion of the cecum in CLP mice had the formation of large abscesses filled with pus. No such abnormalities were found in the CS-injected mice (Figure 3). These data support our conclusion that abnormal chronic production of IL-6 in sepsis survivor mice after CLP is not due to sepsis per se, but rather due to unresolved cecal ligation.

Similar to many other inflammatory cytokines, plasma IL-6 levels are very low (< 20 pg./mL) or undetectable in young healthy mice (17, 24). Though plasma IL-6 levels rapidly increase upon infection or injury, they usually decrease down to the basal level once the initial insult is resolved. Sustained high levels of IL-6 are indicative of unresolved infection and/or poor outcome (17, 18, 24). Previous studies using IL-6 over-expressing transgenic mouse strains have revealed that long-term systemic IL-6 overexpression can cause abnormal phenotypes. These include increased expression of IL-6-inducible hepatic genes, altered B-cell maturation and increased frequency of lymphoma (25), enhanced pulmonary vascular remodeling and pulmonary artery hypertension (26), decreased production of insulin-like growth factor-1 production and subsequent growth impairment (27), and induced muscle atrophy and wasting (28).

We acknowledge a limitation of the present study focusing only on IL-6 as an inflammatory marker instead of characterizing the full cytokine landscape. However, our primary objective was not to perform a global cytokine profiling, but rather to demonstrate the divergence between these two models caused by the surgical artifact. Using IL-6 as a robust and reliable surrogate marker, we effectively identified the persistent inflammatory focus in CLP, which is absent in the CS model. It is highly likely that plasma levels of other inflammatory cytokines are also elevated in mice 1-month after CLP. Therefore, particular caution is required for interpreting any long-term abnormalities if found in mice that survived CLP-induced sepsis.

Prolonged weight loss was more prominent in CLP mice compared to CS mice. The weight loss in CLP and CS mice 1 month after sepsis induction were 5.2 and 0.3%, respectively. Long-term weight loss was most profound in both the CL and CLP groups, and mice in these two groups showed a very similar pattern of weight loss. Compared to these two groups, CLP-D mice showed less severe weight loss, indicating that the weight loss is not only due to cecal ligation or loss of distal cecal function, but also further worsened by the necrosis of the remaining ligated cecum.

Importantly, mice in the CL control group exhibited many abnormalities similar to the CLP sepsis mice at chronic phase. These abnormalities include sustained weight loss, chronic elevation of plasma IL-6 levels, elevated IL-6 gene expression from the ligated cecum (Figure 4), and large abscess formation (Figure 3). Since both CLP and CL mice showed significantly higher systemic inflammation 1 month after sepsis induction as compared to CS mice, the severe chronic inflammation and weight loss after CLP is largely due to the ligation and resulting necrosis of the cecum. Fat loss, as previously characterized in sepsis survivor mice (14), was equally profound in CLP, CLP-D, CS, and CL. Spleen enlargement, also as characterized in sepsis survivor mice (15), was prominent in CL, CLP, CLP-D, and CS. Indeed, except for mortality rates, CL and CLP outcomes were very similar. Thus, many of these acute and chronic CLP-like outcomes in CL mice are likely the consequence of cecal ligation alone. Accordingly, it is likely that some, if not all, mice with CL without puncture developed sepsis, though not as acutely as CLP.

In this study, we included CLP-D mice which underwent debridement of ligated and punctured distal cecum 1 day after CLP surgery. Compared to CL and CLP survivor mice, CLP-D mice had less severe long-term weight loss which was comparable to CS survivors. CLP-D mice also appear to have blunted IL-6 production compared to CL and CLP, although 1 out of 8 mice developed a large cecal abscess with very high IL-6 production (Figures 3B, 4A), and all the mice had truncated or deformed ceca. As mentioned above in Introduction section, cecal debridement after CLP is rarely performed nowadays although it used to be routinely included in a standard CLP procedure in earlier time (19, 20). As the cecal debridement after CLP is a “double surgery” procedure, it may not be allowed to perform in many research institutions. Taken together, the CLP-D model may be considered as a less problematic alternative to CLP, but does not appear to be as promising as CS model for investigating PSS.

There is a possibility that the pronounced acute IL-6 levels observed in the CS model triggered an immunosuppressive or ‘exhausted’ state - often referred to as immunoparalysis (29)—which could contribute to the less IL-6 production in the CS model compared to the CLP model at Day 30. A massive initial inflammatory stimulation can induce long-term immune reprogramming, involving epigenetic changes that dampen subsequent cytokine response (30). This state is a hallmark of Persistent Inflammation, Immunosuppression, and Catabolism Syndrome (PICS) (31). Nevertheless, the very strong chronic IL-6 gene expression specifically within the retained necrotic cecum of CLP mice (Figures 4B,C) indicates that such surgical artifact is the major driver of the chronic inflammation in the CLP model. While immune suppression may occur in both CS and CLP models, the persistent inflammation of necrotic ligated cecum likely overrides the immune regulatory mechanism, resulting in the non-resolving systemic inflammation in the CLP model.

In conclusion, mice that survived CLP-induced sepsis exhibit persistent IL-6 production originated from the surgically ligated portion of the cecum which is abnormally deformed and necrotic. Interpretation of long-term outcomes from CLP-induced sepsis requires cautious consideration of such experimental artifacts. The CS model appears to be better suited for investigating PSS because these cecum problems are absent in this non-surgical model.

## Data Availability

The original contributions presented in the study are included in the article/Supplementary material, further inquiries can be directed to the corresponding author.
