# Supplementary material for: Mouse models for post-sepsis syndrome: a comparison of cecal slurry injection model versus two cecal ligation and puncture models
Source: Front Med (Lausanne). 2026 May 22;13:1774517. doi: 10.3389/fmed.2026.1774517 (PMC13237699; doi:10.3389/fmed.2026.1774517)
Supplement: Supplementary file 1 [file Table_1.DOCX]

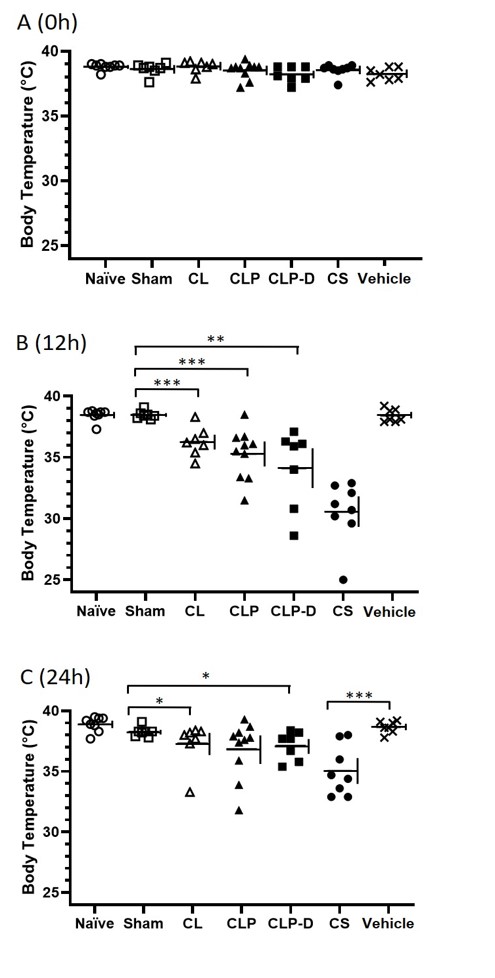
Supplementary Figure S1

**Fig. S1. Body temperature of each individual mouse at 0h (A), 12h (B), and 24h (C) after sepsis induction.** Each symbol represents an individual mouse. Horizontal and vertical lines represent the mean and standard deviation, respectively. All mice that survived for 1 month are included. (A) indicates all mice had normal body temperature before sepsis induction. One-way ANOVA tests show statistical significance in (B) and (C) with P <0.0001 and P = 0.0002, respectively. Pairwise comparisons by t-tests also showed significance. * and *** represent P <0.05 and P <0.001, respectively. The 6h data are shown in the main text (**Fig. 1D**).

***Supplementary Figure S2***


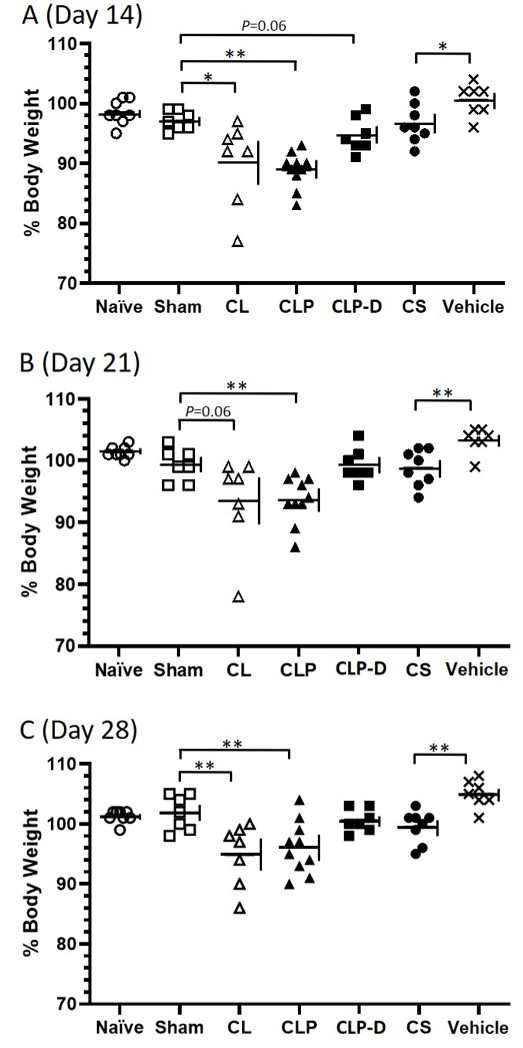


**Fig. S2. Body weight of each individual mouse at Day 14 (A), Day 21 (B), and Day 28 (C) after sepsis induction.** Each symbol represents an individual mouse sample. Horizontal and vertical lines represent the mean and standard deviation, respectively. All mice that survived for 1 month are included. One-way ANOVA tests show P <0.0001 (A-C). Pairwise comparisons by t-tests also showed significance. * and ** represent P <0.05 and P <0.01, respectively. The Day 7 data are shown in the main text (**Fig. 2B**).
